# Cost, and the Varieties of Radiations

**Published:** 1914-03-21

**Authors:** 


### Cost, and the Varieties of Radiations.

At the moment the price of radium is round about £20 per milligram, or over £1,300 per grain. Whether this terrific price is likely to rise still further, or to be maintained, or to fall is a question which affects very closely every researcher in radium and every institution which decides to acquire some of this precious metal. In a published communication a few weeks ago, the director of research in the cancer laboratories of the Middlesex Hospital said the price was artificially inflated. Commenting on that assertion (The Hospital, January 17, page 410) we pointed out that a more important factor in the rising price was the keen competition which exists for radium, together with the extremely limited supply. This demand is increasing faster than the extraction of radium from its ores, and so the price is still rising. Undoubtedly there has been a good deal of manipulation by a "ring" as well; but this is not the main factor in the market value of radium.

Radium can never be cheap: the mere processes of extraction from the ores are so lengthy and expensive, and the ultimate result so exiguous, that unless deposits of radium-containing ores are found much stronger in radium than any at present known, it will always be exceedingly dear. Will it, however, be cheaper than it is now? We can well imagine that a good many boards of governors would be glad of an answer to that question. Sir Frederick Treves, speaking at the Medical Society of London on March 9, mentioned some quite new discoveries of radium-containing earths, and prophesied that within a reasonable time the price will be down to £10 per milligram—half of what it stands at just now. It is also to be remembered that during extraction several very valuable by-products are obtained; so that the manufacturers of radium, costly though their plant and processes are, can in reality produce radium at a profit at a very much lower price than they now ask and obtain. So that cheaper radium does not at all mean bankruptcy for the manufacturers, and consequent cessation of production.

Radium itself gives out radiations of two distinct kinds, known as $\alpha$ and $\beta$ radiations. Radium emanation also gives off $\alpha$-rays, and produces in the process a solid substance known as radium A. Radium A also gives off $\alpha$-rays, and is changed into radium B and then into radium C. During this last change both $\alpha$ and $\beta$ rays are produced, and from radium C itself there arise three kinds of rays, $\alpha$, $\beta$, and $\gamma$. Next there is formation from radium C of another product, radium D, which emits $\beta$-rays of low velocity. Then follows radium E, which gives $\beta$ and $\gamma$ radiations, and radium F, which gives $\alpha$ radiations. This latter body is believed to be identical with another metal, known as polonium.

The physical characters of these three different kinds of rays need not detain us now. Suffice it that the $\alpha$ and $\beta$ rays are believed to consist of actual particles or atoms projected from the radium as a source, and that the exact nature of the $\gamma$-rays is still a matter of debate. The $x$-rays are similar to the $\gamma$-rays of radium, but are not exactly the same. It is supposed that the good effects of both $x$-ray treatment and of radium are due to the $\gamma$-rays; but even this proposition is not absolutely certain, and an American worker has lately upheld the superiority of the $\beta$ radiations. In radium therapeutics, whether emanation or radium is being used, screens of various thicknesses and various materials are used in order to filter off those rays which it is not desired to utilise. Broadly speaking it is the $\gamma$-rays which are most employed. Screens of aluminium, brass, lead, silver, platinum, glass, rubber, horn, and other substances are used; and to protect the healthy tissues round about those diseased parts upon which action is sought for the former are covered with protectives, such as black paper, lint, gauze, rubber, and suchlike. The details as to thickness and nature of screen are matters of taste about which individual workers hold different opinions. One very important point is that the primary radiations in passing through metal screens may set up secondary radiations, not always of the same type as those which excite them; and in consequence of this there is a tendency to use more and more for screening purposes those metals a very thin sheet of which will prove effective for this purpose, especially aluminium and silver.

